# Predictors of early childhood HIV testing among children of sex workers living with HIV in Cameroon

**DOI:** 10.1186/s12889-019-6812-3

**Published:** 2019-05-29

**Authors:** Amrita Rao, Sheree Schwartz, Serge C. Billong, Anna Bowring, Ghislaine Fouda, Flavien Ndonko, Iliassou Njindam, Daniel Levitt, Anne-C. Bissek, Oudou Njoya, Stefan Baral

**Affiliations:** 1 Department of Epidemiology, Johns Hopkins Bloomberg School of Public Health, 615 N. Wolfe St., Baltimore, MD 21205 USA; 2 University of Yaoundé, Yaoundé, Cameroon; 3 CARE Cameroun, Yaoundé, Cameroon; 4 CARE USA, New York, NY USA; 5 Faculty of Medicine and Biomedical Sciences, University of Yaoundé, Yaoundé, Cameroon; 6 Division of Operational Research in Health, Ministry of Public Health, Yaoundé, Cameroon

**Keywords:** Female sex workers, Vertical transmission of HIV, Early childhood HIV testing, Antenatal care attendance, Cameroon

## Abstract

**Background:**

Despite recent progress, there exist gaps in the prevention of vertical HIV transmission program access and uptake in Cameroon. Female sex workers (FSW), many of whom are mothers, are disproportionately affected by HIV and have specific barriers to HIV testing and treatment access. Testing for HIV-exposed infants is crucial in monitoring for incident infection and timely intervention. This study explores the level of early childhood testing and also associations between antenatal care (ANC) attendance and other factors and early childhood HIV testing among FSW in Cameroon.

**Methods:**

FSW were recruited to participate in an integrated biobehavioral survey in Cameroon between December 2015 and October 2016. Women were included in these analyses if they were living with HIV and had at least one living child. Both univariate and multivariable logistic regression were used to look at predictors of a child being tested for HIV before age five.

**Results:**

A total of 481/2255 FSW were eligible for these analyses as they were HIV seropositive and had at least one living child at the time of the study. Women included in these analyses had a median age of 35(IQR 30–41). Nearly 70% reported none of their children had been tested for HIV before age five (326/481), and 3.5%(17/481) reported one or more of their children had been diagnosed with HIV. ANC attendance (adjusted OR 2.12, 95% CI: [1.02, 4.55]), awareness of HIV status (aOR 3.70[2.30, 5.93]), pregnancy intentions (aOR 1.89[1.16, 3.08]), and higher education (aOR 2.17[1.01, 4.71]) were all independently associated with increased odds of women having a greater proportion of children tested for HIV before age five. Regional differences in early childhood testing were also observed.

**Conclusion:**

Vertical transmission of HIV remains a challenge in Cameroon, and HIV testing among children of FSW living with HIV was very low. ANC attendance and promotion of the mother’s health were associated with increased child HIV testing. For women at high risk of HIV and for whom engagement in the health system is low, strategies to promote and ensure ANC attendance are essential for their health and the health of their children.

**Electronic supplementary material:**

The online version of this article (10.1186/s12889-019-6812-3) contains supplementary material, which is available to authorized users.

## Background

There are an estimated 35,000 incident HIV infections each year in Cameroon [1]. Women, especially women of reproductive age, account for 60% of the 620,000 people living with HIV nationally, with an HIV prevalence of approximately 6% [2]. Pediatric acquisition of HIV remains a major challenge, and vertical transmission is responsible for just over 5% of new infections annually in Cameroon [3]. Despite some recent improvements, a total of 4100 infants were diagnosed with HIV during infancy in Cameroon in 2015, and regional estimates suggest that the median life expectancy for these children is 2 years [4]. These estimates may underrepresent the number of children living with HIV in Cameroon given that infant testing rates are low, with only 30% of infants born to women living with HIV in Cameroon receiving a virological test by 2 months of age [5].

Children of sex workers have been noted to be at increased risk of acquiring HIV through vertical transmission due to the greater burden of HIV and pervasive structural barriers to the access and uptake of HIV treatment and care experienced by their mothers [6]. There are an estimated 112,000 female sex workers (FSW) in Cameroon, and many of these women are mothers [7]. Although the majority of FSW are mothers and are much more likely to be living with HIV, experiencing a prevalence of HIV closer to 37%, little is known about their prevention of mother to child transmission (PMTCT) needs [7]. This includes unmet need for family planning, strategies for safer conception, engagement in antenatal care (ANC), and early childhood health outcomes. The increased risk among children of sex workers is not specific to Cameroon; one study of the cause of child mortality among children of FSW in Cambodia found that HIV was the primary reported cause of death, accounting for 36% of deaths among children under five [8]. However, there remains limited data available and a need to better understand this important and often overlooked population [6].

Out of 22 countries prioritized by the World Health Organization (WHO) as part of the Global Plan towards the elimination of new HIV infections among children, Cameroon was among the top 10 in terms of PMTCT investment priority [9]. Despite significant scale-up of programs and a 49% decline in new HIV infections among children between 2009 and 2015, the goal of reducing the vertical transmission rate to 5% or less among breastfeeding women and to 2% or less among non-breastfeeding women has yet to be achieved in Cameroon [9]. As of 2015 in Cameroon, just over 80% of pregnant women living with HIV received antiretrovirals for PMTCT [9]. In addition, there are marked disparities in access to services by region, with ANC attendance ranging from 20% to over 60%, with the greatest proportion of those who do not attend any ANC visits in the Centre, Far-North, Littoral, Adamawa, North-East, West, and South-West regions [10]. Given the burden, understanding and addressing the needs of FSW and their children could have a potentially large impact on reduction of vertical transmission of HIV in Cameroon and reaching the goals put forward as part of the Global Plan.

The purpose of these analyses is to gain insights into understanding PMTCT needs of FSW and their children in five cities in Cameroon (Yaoundé, Douala, Bertoua, Bamenda, and Kribi) between 2015 and 2016 through examining levels of early childhood testing among children of FSW living with HIV and studying the relationship between ANC attendance and the proportion of a woman’s children tested before the age of five.

## Methods

### Study design, population and procedures

Data are from a cross-sectional integrated bio-behavioral survey (IBBS) assessing knowledge, attitudes, and behavioral and biological risk factors associated with HIV infection among FSW in five cities across Cameroon. The objective of the IBBS was to translate findings into programmatic interventions and to propose strategies for reducing burden of disease. FSW were recruited for this study using respondent-driven sampling, which is a strategy employed when individuals in the target population are hard-to-reach and when no known sampling frame exists [11]. Methods for respondent-driven sampling have been described previously [11]. A total of 6 seeds [Yaoundé: 2, Douala: 1, Bertoua:1, Bamenda:1, Kribi:1] resulted in the recruitment of 2255 participants. Participants received 2000 Central African Franc (XAF) for their participation and 500 XAF, approximately 0.85 USD, for every eligible participant they recruited for up to three recruits. FSW were recruited from Yaoundé, Douala, Bertoua, Bamenda, and Kribi between December 2015 and October 2016.

Eligible participants were women who were 18 years or older, assigned the female sex at birth, and reported selling sex as a primary source of income in the last 12 months. Women were included in the current analysis if they were HIV seropositive and had at least one living child at the time of the study. All study participants completed written informed consent in either English or French prior to enrolling in the study. Ethical approval for the study was obtained from the National Research Ethics Committee in Cameroon, along with the Johns Hopkins School of Public Health Institutional Review Board.

Following provision of written informed consent, women completed a 45 to 60-min interviewer-administered questionnaire, answering questions related to demographics, sexual and reproductive health history, human rights abuses, and utilization of HIV prevention and treatment services. Blood draws and HIV testing were conducted according to national procedures. For HIV testing, all women were given a first-line rapid test: Alere Determine™ HIV-1/2 Ag/Ab Combo Rapid Test Kit. If nonreactive, the test result for that participant was recorded as HIV-negative. If reactive, a second rapid test, OraSure OraQuick® HIV- 1/2 was administered. If reactive for the OraQuick HIV- 1/2 test, the test result for that participant was recorded as HIV- positive. If nonreactive test result for the participant was recorded as HIV-negative. Pretest and posttest counseling was provided per national guidelines. Those testing positive for HIV were referred to treatment and care facilities for further management.

### Outcome

The primary outcome examined in these analyses was the proportion of a woman’s children tested for HIV before the age of five. The proportion of children tested was calculated by taking the number of children who were tested for HIV before age five [“How many of your children were tested for HIV before they were 5 years old?”] and dividing it by the total number of living children a woman reported having [“How many of your biological children are currently living?”]. Both questions were answered via self-report.

### Covariates of interest

The main independent variable of interest was ANC attendance during a woman’s last pregnancy, defined as “yes, attended” or “no, did not attend.” Women were asked “The last time that you were pregnant, did you go to the clinic for antenatal care during your pregnancy? This is care that you receive from health care providers during your pregnancy to ensure that you and your baby are well, and to promote a healthy pregnancy.” Other covariates of interest included site of recruitment, age, highest level of education completed, income, years selling sex, future pregnancy intentions, parity, network size, and awareness of HIV status, all of which, other than site of recruitment, were collected via self-report. Future pregnancy intentions were dichotomized and women were asked “Do you plan to or hope to have more children in the future?” To get at network size, women were asked “how many female sex workers have you met in the past week?”

### Statistical analyses

Among FSW living with HIV and who had at least one living child, characteristics were compared by recruitment site using Fisher’s exact tests. Logistic regression was used to examine the associations between ANC attendance and other predictors and the odds of a child having been tested for HIV before the age of five. Both crude and adjusted associations were examined. Covariate predictors were selected for inclusion in the final multivariable model based on a consideration of both statistically significant associations with the outcome in univariate models (*p* < 0.10) and a priori hypothesized relationships. As all variables had less than 1% missing data, complete case analysis was utilized.

### Sensitivity analysis

The association between ANC attendance and the odds of a child having been tested for HIV before the age of five was also examined among a restricted sample of women who had children 5 years old or younger at the time of the study. By restricting to women with children under five, a more proximal measure of risk was assessed given that testing guidelines are rapidly evolving and children born within the past 5 years may reflect current child testing trends.

## Results

Of the 2255 FSW recruited to participate in the primary study, 1889 (84%), were mothers, and about 24% [95% CI: 23, 26%] were living with HIV (547/2255). Of the 2255 women recruited for the primary study, 1708 were excluded from these analyses as they were not living with HIV. An additional 66 women living with HIV were excluded as they were not mothers. Among the 481 FSW included in these analyses, 84% attended ANC during their last pregnancy (406/481), and 52% had knowledge of their HIV seropositive status (251/481). Women had a median a two children (IQR: 1–3). Nearly 70% of women reported none of their children had been tested for HIV before age five (326/481), and 17 (3.5%) reported at least one of their children were previously diagnosed with HIV.

Characteristics of study participants by site of recruitment are described (Table 1**).** There was a relatively equal distribution of women across sites, with about one quarter of included women having been recruited from Yaoundé. Women recruited from Douala were significantly less likely to have attended ANC during their last pregnancy, while women recruited from Bertoua and Bamenda were significantly more likely to have attended (*p* < 0.001). The median age was 35 years (IQR: 30–41). Younger women were more likely to have been recruited from Bertoua and Kribi, while older women were more likely to have been recruited from Bamenda (*p* < 0.001). More than 45% of women had completed primary school or less (217/481). Close to 70% of women reported making less than or equal to 100,000 XAF a month, or about 6 USD per day, with lower-income women being more likely to be recruited from Bamenda (*p* < 0.001). Women reported having sold sex for a median of 5 years (IQR: 3–10). About 20% of women did not report any form of contraception other than condoms (84/481), with those attending ANC being more likely to be using long-acting reversible contraceptives than those not attending (*p* < 0.01). The median age of a woman’s youngest child was 8 years (IQR: 4–13).

**Table 1:** Characteristics of female sex workers living with HIV by site of recruitment in Cameroon, 2015–2016 (*n* = 481)

	Overall *n* = 481	Yaoundé *n* = 120	Douala *n* = 122	Bertoua *n* = 61	Bamenda *n* = 101	Kribi *n* = 77	*p*-value
	n (%)	n (%)	n (%)	n (%)	n (%)	n (%)	
ANC attendance at last pregnancy							**< 0.001**
No, did not attend	75 (15.6%)	20 (16.7%)	35 (28.7%)	4 (6.6%)	3 (3.0%)	13 (16.9%)	
Attended	406 (84.4%)	100 (83.3%)	87 (71.3%)	57 (93.4%)	98 (97.0%)	64 (83.1%)	
Age, years							
18–24	43 (8.9%)	6 (5.0%)	6 (4.9%)	15 (24.6%)	6 (5.9%)	10 (13.0%)	**< 0.001**
25–34	182 (37.9%)	46 (38.3%)	39 (32.0%)	28 (45.9%)	29 (28.7%)	40 (52.0%)	
35+	256 (53.2%)	68 (56.7%)	77 (63.1%)	18 (29.5%)	66 (65.4%)	27 (35.0%)	
Education completed^a^							
Primary or less	217 (45.2%)	38 (31.7%)	60 (49.2%)	22 (36.7%)	68 (67.3%)	29 (37.7%)	**< 0.001**
Some secondary	237 (49.4%)	74 (61.7%)	57 (46.7%)	36 (60.0%)	25 (24.8%)	25 (58.4%)	
Secondary or more	26 (5.4%)	8 (6.6%)	5 (4.1%)	2 (3.3%)	8 (7.9%)	3 (3.9%)	
Monthly income							
≤ 100,000 XAF	329 (68.4%)	87 (72.5%)	47 (38.5%)	39 (63.9%)	99 (98.0%)	57 (74.0%)	**< 0.001**
> 100,000 XAF	152 (31.6%)	33 (27.5%)	75 (61.5%)	22 (36.1%)	2 (2.0%)	20 (26.0%)	
Years selling sex							
0–2 years	109 (22.7%)	24 (20.0%)	24 (19.6%)	21 (34.4%)	10 (9.9%)	30 (39.0%)	**< 0.001**
3–9 years	228 (47.4%)	60 (50.0%)	70 (57.4%)	20 (47.5%)	35 (34.7%)	34 (44.1%)	
10+ years	144 (29.9%)	36 (30.0%)	28 (23.0%)	11 (18.0%)	56 (55.4%)	13 (16.9%)	
Awareness of HIV status							
No awareness of status	230 (47.8%)	56 (46.7%)	49 (40.2%)	36 (59.0%)	34 (33.7%)	55 (71.4%)	**< 0.001**
Aware of status	251 (52.2%)	64 (53.3%)	73 (59.8%)	25 (41.0%)	67 (66.3%)	22 (28.6%)	
Number of living children							
One	132 (27.4%)	28 (23.3%)	43 (35.3%)	13 (21.3%)	21 (20.8%)	27 (35.0%)	**0.02**
Two	145 (30.2%)	47 (39.2%)	28 (23.0%)	19 (31.2%)	27 (26.7%)	24 (31.2%)	
More than two	204 (42.4%)	45 (37.5%)	51 (41.7%)	29 (47.5%)	53 (52.5%)	26 (33.8%)	
Future pregnancy intentions							
None	267 (55.5%)	55 (45.8%)	81 (66.4%)	36 (59.0%)	68 (67.3%)	27 (35.1%)	**< 0.001**
Want more children	214 (44.5%)	65 (54.2%)	41 (33.6%)	25 (41.0%)	33 (32.7%)	50 (64.9%)	
Network size^b^							
*Mean*	13.4	14.4	20.3	13.8	9.7	5.3	**< 0.001**
*Standard deviation*	22.3	25.5	29.7	19.8	12.5	6.3	

^a^Data missing on education (*n* = 1). Bolded results represent results that are statistically significant at least at the *p* < 0.05 level

^b^Significant differences calculated using Bartlett’s test for equal variances. To get at network size, women were asked “how many female sex workers have you met in the past week?”

### Early childhood testing

Antenatal care attendance was associated with an increased odds of a child being tested for HIV before age 5 compared to not having attended (OR: 4.40, 95% CI 2.01, 9.61) (Table 2). Having been recruited from Douala (OR: 0.18, 95% CI 0.09, 0.34) or Kribi (OR: 0.45, 95% CI 0.24, 0.84) was associated with a decreased odds of HIV child testing before the age of five, while having been recruited from Bamenda (OR: 4.20, 95% CI 2.69, 6.53) was associated with an increased odds of HIV child testing all when compared with those recruited from Yaoundé. Completion of secondary or higher education compared to primary or less (OR: 2.98, 95% CI 1.42, 6.25), future pregnancy intentions compared to no future intentions (OR: 1.52, 95% CI 1.03, 2.24, and awareness of HIV status compared to no awareness (OR: 3.20, 95% CI 2.91, 4.90) were all significantly associated with increased odds of having a child tested for HIV before the age of five in univariate models. In the multivariable model including site of recruitment, ANC attendance at last pregnancy, age, education level, pregnancy intentions, and awareness of HIV status, variables remaining statistically significantly associated with a higher odds of early childhood testing included site of recruitment, ANC attendance, future pregnancy intentions, awareness of HIV status, and higher education. Having been recruited from Bamenda remained significantly associated with higher odds, while having been recruited from Douala and Kribi remained significantly associated with lower odds compared to having been recruited from Yaoundé.

**Table 2:** Crude and adjusted odds ratios of living children being tested for HIV before the age of five among female sex workers living with HIV in Cameroon, 2015–2016 (*n* = 477)^a^

	Odds Ratio [95% CI]	Adjusted Odds Ratio [95% CI]
Site of recruitment		
Yaoundé	REF	REF
Douala	0.18 [0.09, 0.34]***	0.20 [0.10, 0.41]***
Bertoua	1.17 [0.66, 2.05]	1.02 [0.50, 2.06]
Bamenda	4.20 [2.69, 6.53]***	2.34 [1.30, 4.22]**
Kribi	0.45 [0.24, 0.84]*	0.46 [0.22, 0.98]*
Antenatal care attendance		
Did not attend	REF	REF
Attended during last pregnancy	4.40 [2.01, 9.61]***	2.12 [1.02, 4.55]*
Age		
18–24	REF	REF
25–34	1.13 [0.76, 1.67]	0.71 [0.31, 1.61]
35+	0.79 [0.54, 1.16]	0.56 [0.24, 1.30]
Education		
Primary or less	REF	REF
Some secondary	0.75 [0.51, 1.10]	0.92 [0.57, 1.49]
Secondary or more	2.98 [1.42, 6.25] **	2.17 [1.01. 4.71]*
Years selling sex		
0–2 years	REF	
3–9 years	0.90 [0.61, 1.33]	
10+ years	1.34 [0.89, 2.03]	
Pregnancy Intentions		
No future intentions	REF	REF
Has future intentions	1.52 [1.03, 2.24]*	1.89 [1.16, 3.08]*
Number of living children		
One	REF	
Two	0.84 [0.55, 1.29]	
More than two	1.09 [0.74, 1.60]	
Awareness of HIV status		
No awareness	REF	REF
Aware of HIV status	3.20 [2.91, 4.90]***	3.70 [2.30, 5.93]***
Network size	0.99 [0.97, 1.01]	

Odds ratios were estimated using logistic regression. ^a^Five women were excluded from analysis due to missing data on proportion of children tested before age five (*n* = 4) or highest level of education (*n* = 1). *Statistically significant at *p* < 0.05; **p < 0.01; ***p < 0.001. Bolded results represent results that are statistically significant at least at the *p* < 0.05 level

### Sensitivity analyses

In the planned sensitivity analyses restricted to the subsample of women with at least one child ≤5 years at the time of the study (*n* = 180), no qualitative differences in results emerged when assessing the association between ANC attendance and the odds of a child tested for HIV before the age of five (results not shown).

## Discussion

These analyses of the reproductive and maternal health outcomes among FSW mothers living with HIV and their children highlight the need to prioritize the health of the mother through early and continued engagement in testing and care. In this group of FSW living with HIV, more than half of women were unaware of their infection status and just over one in six women reported not attending ANC during their last pregnancy. Nearly 70% of FSW living with HIV reported that none of their children had been tested for HIV before the age of five, and about 4% of women reported at least one of their children had been previously diagnosed with HIV. ANC attendance, in conjunction with site-specific differences, higher level of completed education, future pregnancy intentions, and awareness of HIV status, proved important correlates of early childhood testing.

Despite the need for HIV testing among children of mothers living with HIV - only 30% of FSW mothers in this study reported that any of their children had been tested for HIV before age five - little is known about engagement in testing and care or the health outcomes of children of FSW [5]. Existing data focus primarily on the challenges of motherhood and additional vulnerability for the woman, especially as it relates to negotiating power [12–15]. In southern Tanzania, it was found that sex workers who were mothers reported they were more likely to take on additional clients and accept more money for condomless sex [12]. Despite knowledge of risks, women felt that their choices were constrained given that they had children to support [12]. Another study in southern India similarly found that women who reported three or more children were significantly less likely to report consistent condom use and more likely to report condomless commercial sex [13]. Despite these data, there is little available data which focus on testing and health outcomes of children of FSW and ways to mitigate vertical transmission in this vulnerable group.

Awareness of HIV status in these mothers was very low, and when looking at maternal engagement in testing and care in the broader context, there exist clear differences between FSW and other reproductive aged women. For example, in a single study looking at a combined retrospective cohort of 1866 mothers and a prospective cohort of 150 mothers in Cameroon, the overall prevalence of maternal HIV infection was 5% [16]. Even with limited reported knowledge of modes of vertical transmission, all of those testing positive in this group of women were aware of their status, compared with just 52% of FSW in this study [16]. ANC attendance among other women of reproductive age in Cameroon has been shown to be quite high, with one study reporting that 85% of women attended at least one ANC visit in 2011–96% in urban areas and 76% in rural areas - compared to 84% of FSW in this study [17]. Given both the disproportionate burden of HIV faced by FSW, 24% in this study of FSW compared with just over 5% among other women of reproductive age [18], along with the lower levels of both ANC coverage and awareness of HIV status for these women, a critical target for intervention may be missed if sex workers and their children are not made a priority in efforts to scale-up provision of services.

The observed site-specific differences show that looking at national estimates and data in the aggregate may not give an accurate picture of the disparities in maternal health outcomes and engagement in care by region. Differences in region-specific estimates of childhood health indicators have previously been observed, supporting the findings that early childhood testing rates may be region-specific [19]. In this study among FSW, lower odds of early childhood testing were observed in Douala and Kribi compared with Yaoundé. In these same areas in a larger Demographic and Health Survey, there was a higher prevalence of children under 5 years classified as malnourished and anemic [19]. FSW in Bamenda reported high levels of both ANC attendance and early childhood testing. This success in the North West region of the country may reflect the recent scale-up of PMTCT programs [18]. An increase in funding allowed for an increase in geographic coverage and scope of PMTCT programs, including community mobilization efforts, the creation of a PMTCT technical working group, greater staff capacity, along with other efforts [18]. According to one report, PEPFAR-supported PMTCT services led to increases in ANC attendance and the proportion of pregnant women tested for HIV [1]. The construction and provision of subnational estimates and further research to understand differences by region will be crucial for providing focused and effective interventions, for both FSW and the general population.

There are limitations to these analyses. Adjustments for respondent-driven sampling were not conducted as we analyzed the subset of HIV positive mothers in the study population and thus recruitment chains were interrupted and connections needed to understand network ties and apply adjustments were not made. Women were asked about ANC attendance during their last pregnancy and little can be said about whether or not a woman attended ANC at each of her other pregnancies and how this relates to the health outcomes of any specific child. Despite this limitation, ANC attendance during the most recent pregnancy speaks to a woman’s engagement in care during pregnancy, a critical period for prevention of vertical transmission. Additionally, the primary analyses do not consider era-effects of changing testing guidelines. We did, however, attempt to understand these era-effects by restricting the sample to women who had children in the last 5 years in sensitivity analyses, and no appreciable differences were seen when looking at the primary relationships of interest. Furthermore, given the structure of the questions, we were not able to ascertain the testing status of deceased children and to what extent mortality may have been related to HIV.

## Conclusions

The high overall prevalence of maternal HIV and low awareness of HIV status in this group of FSW highlight the need to consider different strategies to strengthen current PMTCT intervention strategies. Taking into consideration that many FSW are mothers and promoting the health of the mother through early engagement in testing and care for herself and her children may improve maternal and child health outcomes. It will be important to integrate child-specific services, including HIV testing during early childhood, into existing FSW services, and site-specific differences should be considered in choosing appropriate interventions for the given context. Given the barriers to care, accommodating hard-to-reach women in national PMTCT programs, including sensitization and stigma mitigation of health care workers to sex work, promotion of both ANC attendance and HIV testing among FSW, and active referrals from community-based organizations working with FSW to ANC, may help reduce gaps in PMTCT access and uptake. Leveraging existing outreach workers and case managers from community-based organizations may help facilitate better linkage to care at community health centers. ANC services for at-risk women can be bolstered with intensified HIV testing services and information on prevention of vertical transmission to improve health outcomes for both mother and child.

A French translation of this article has been included as Additional file 1.

A Portuguese translation of the abstract has been included as Additional file 2.

## Additional files

Additional file 1: Translation of this article into French (PDF 249 kb)

Additional file 2: Translation of the abstract of this article into Portuguese (PDF 101 kb)

## Abbreviations

ANC: Antenatal Care
FSW: Female Sex Workers
HIV: Human Immunodeficiency Virus
IBBS: Integrated bio-behavioral survey
PMTCT: Prevention of mother to child transmission
USD: U.S. Dollar
WHO: World Health Organization
XAF: Central African Franc
