# Supplementary material for: Predictors of early childhood HIV testing among children of sex workers living with HIV in Cameroon
Source: BMC Public Health. 2019 May 29;19(Suppl 1):602. doi: 10.1186/s12889-019-6812-3 (PMC6538542; doi:10.1186/s12889-019-6812-3)
Supplement: Supplementary file 1 — Translation of this article into French (PDF 249 kb) [file 12889_2019_6812_MOESM1_ESM.pdf]

BMC Public Health 2019, 19 (Suppl 1) [translated from the original publication]

Indicateurs de dépistage du VIH dans la petite enfance parmi les enfants des travailleuses du sexe porteuses du VIH au Cameroun

Amrita Rao[1], Sheree Schwartz[1], Serge C. Billong[2], Anna Bowring[1], Ghislaine Fouda[3], Flavien Ndonko[3], Iliassou Njindam[1], Daniel, Levitt[4], Anne-C. Bissek[5,6], Oudou Njoya[5], Stefan Baral[1]

[1]Johns Hopkins Bloomberg School of Public Health, Epidemiology, Baltimore, Maryland, United States
[2]University of Yaoundé, Yaoundé, Cameroon
[3]CARE Cameroun, Yaoundé, Cameroon
[4]CARE USA, New York, New York, United States
[5]University of Yaoundé, Faculty of Medicine and Biomedical Sciences, Yaoundé, Cameroon
[6]Ministry of Public Health, Division of Operational Research in Health, Yaoundé, Cameroon

Auteur-ressource : Amrita Rao, ScM, Department of Epidemiology, Johns Hopkins Bloomberg School of Public Health, 615 N. Wolfe St. Baltimore, MD, 21205, adresse électronique : arao24@jhu.edu.

Adresses électroniques des co-auteurs : Sheree Schwartz sschwartz@jhu.edu, Serge Billong sergebillong@yahoo.fr, Anna Bowring abowrin1@jhu.edu, Ghislane Fouda fouda@carecameroun.org, Flavien Ndonko ndonko@carecameroun.org, Iliassou Mfochive Njindam imfochi1@jhu.edu, Daniel Levitt daniel_levitt@me.com, Anne Bissek annezkbissek@yahoo.fr, Oudou Njoya oudou_nj@yahoo.fr, Stefan Baral sbaral@jhu.edu

RÉSUMÉ

Contexte Malgré les progrès récents, il existe des lacunes dans la prévention de la transmission verticale du VIH, l'accès et le recours au programme au Cameroun. Les travailleuses du sexe (TS), dont beaucoup sont mères, sont touchées de manière disproportionnée par le VIH et doivent passer des barrières spécifiques à l'accès au dépistage et aux traitements. Le dépistage des nourrissons exposés au VIH est crucial dans la surveillance de l'infection et pour une intervention en temps opportun. L'étude explore le niveau de dépistage chez les jeunes enfants et également les associations entre le recours aux soins prénatals (SPN) et d'autres facteurs, et le dépistage du VIH chez les jeunes enfants parmi les TS au Cameroun.

Méthodes Des TS ont été recrutées pour participer à une enquête biocomportementale intégrée au Cameroun, entre décembre 2015 et octobre 2016. Les femmes ont été incluses dans ces analyses, dès lors qu'elles étaient porteuses du VIH et avaient au moins un enfant en vie. On a utilisé à la fois la régression logistique à variables simples et multiples pour considérer les indicateurs de dépistage du VIH d'un enfant avant l'âge de cinq ans.

Résultats Un total de 481/2 255 TS étaient éligibles à ces analyses car elles étaient séropositives au VIH et avaient au moins un enfant en vie au moment de l'étude. Les femmes incluses dans ces analyses avaient un âge médian de 35 ans (EI 30-41). Presque 70 % ont rapporté qu'aucun de leurs enfants n'avait été dépisté pour le VIH avant l'âge de cinq ans (326/481), et 3,5 % (17/481) ont rapporté qu'un ou plusieurs de leurs enfants avaient été diagnostiqués séropositifs. Le recours aux SPN (RC corrigé 2,12, 95 % IC : [1,02, 4,55]), la conscience de son statut VIH (RCc 3,70[2,30, 5,93]), les intentions de grossesse (RCc 1,89 [1,16, 3,08]), et un niveau d'éducation plus élevé (RCc 2,17[1,01, 4,71]) sont apparus comme des facteurs

indépendants associés à une probabilité accrue que les femmes aient une plus grande proportion d'enfants testés pour le VIH avant l'âge de cinq ans. Des différences régionales ont également été observées concernant le dépistage des jeunes enfants.

Conclusion La transmission verticale du VIH reste un défi au Cameroun, et le dépistage du VIH parmi les enfants des TS porteuses du VIH était très faible. Le recours aux SPN et la promotion de la santé maternelle sont associés à des taux accrus de dépistage du VIH chez les enfants. Pour les femmes à risque élevé de VIH peu engagées dans le système de santé, les stratégies pour promouvoir et assurer le recours aux SPN sont essentielles pour leur santé et celle de leurs enfants.

Mots clés : travailleuses du sexe, transmission verticale du VIH, dépistage du VIH chez les jeunes enfants, recours aux soins prénatals, Cameroun

CONTEXTE

On estime à 35 000 le nombre annuel d'infections au VIH au Cameroun[1]. Les femmes, en particulier en âge de procréer, représentent 60 % des 620 000 personnes porteuses du VIH, avec un taux de prévalence du VIH d'environ 6 %[2]. La contamination pédiatrique par le VIH reste un défi majeur et la transmission verticale est responsable de plus de 5 % des nouvelles infections chaque année au Cameroun[3]. Malgré des améliorations récentes, un total de 4 100 nourrissons ont été diagnostiqués séropositifs au VIH au Cameroun en 2015, et des estimations régionales suggèrent que l'espérance de vie médiane de ces enfants est de deux ans [4]. Ces estimations peuvent sous-représenter le nombre d'enfants porteurs du VIH au Cameroun, étant donné que les taux de dépistage sont faibles, avec seulement 30 % des nourrissons de mères séropositives soumises à un test virologique avant l'âge de deux mois [5].

Les enfants des travailleuses du sexe présentent un risque accru d'infection au VIH par transmission verticale, en raison des obstacles structurels généralisés dans l'accès et le recours aux traitements anti-VIH pour leurs mères[6]. On estime à 112 000 le nombre de travailleuses du sexe au Cameroun, et beaucoup de ces femmes sont mères[7]. Bien que la majorité des TS soient mères et plus susceptibles d'être porteuses du VIH, avec un taux de prévalence du VIH proche de 37 %, on en sait peu sur leurs besoins en termes de prévention de la transmission de

la mère à l'enfant (PTME)[7]. Ceci inclut le besoin non couvert de planning familial, les stratégies pour une conception plus sûre, l'engagement dans les soins prénatals (SPN) et les problèmes de santé de la petite enfance. Le risque accru parmi les enfants des travailleuses du sexe n'apparaît pas comme spécifique au Cameroun ; une étude sur la cause de la mortalité infantile parmi les enfants des TS au Cambodge indique que le VIH est la première cause de décès rapportée, avec 36 % des décès parmi les enfants de moins de cinq ans[8]. Toutefois, les données disponibles sont limitées pour mieux comprendre cette population importante et souvent négligée[6].

Sur 22 pays priorisés par l'Organisation mondiale de la Santé (OMS) dans le plan mondial pour l'élimination des nouvelles infections au VIH chez les enfants, le Cameroun faisait partie du top 10 prioritaire en termes d'investissements dans la PTME [9]. Malgré l'amplification significative des programmes et une baisse de 49 % du nombre de nouvelles infections au VIH parmi les enfants entre 2009 et 2015, l'objectif de réduire le taux de transmission verticale à 5 % ou moins parmi les femmes allaitantes et à 2 % ou moins parmi les femmes non-allaitantes n'est pas encore atteint au Cameroun[9]. En 2015 au Cameroun, un peu plus de 80 % des femmes enceintes porteuses du VIH ont reçu des antirétroviraux pour la PTME[9]. De plus, on observe des disparités régionales marquées dans l'accès aux services, avec un recours aux SPN allant de 20 % à plus de 60 %, avec la proportion la plus élevée de visites de SPN non effectuée dans les régions Centre, Extrême-Nord, Littoral, Adamawa, Nord-Est, Ouest et Sud-Ouest[10]. Considérant l'incidence, la compréhension et la gestion des besoins des TS et de leurs enfants pourraient avoir un fort impact dans la réduction de la transmission verticale du VIH au Cameroun et pour atteindre les objectifs du plan mondial.

L'objectif de ces analyses est d'obtenir les renseignements permettant de comprendre les besoins des TS et de leurs enfants pour la PTME dans cinq villes du Cameroun (Yaoundé, Douala, Bertoua, Bamenda et Kribi) entre 2015 et 2016, en examinant les niveaux de dépistage dans la petite enfance parmi les enfants des TS porteuses du VIH et en étudiant le lien entre le recours aux SPN et la proportion des enfants testés avant l'âge de cinq ans.

MÉTHODES

Conception de l'étude, population et procédures

Les données proviennent d'une enquête transversale biocomportementale intégrée (EBCI) évaluant les connaissances, les attitudes, ainsi que les facteurs de risque comportementaux et biologiques associés à l'infection par le VIH parmi les TS dans cinq villes du Cameroun. L'objectif de l'EBCI était de traduire les résultats dans des interventions programmatiques et de proposer des stratégies permettant de réduire la charge de la maladie. Les TS ont été recrutées pour cette étude sur la base d'un échantillonnage en fonction des répondants qui constitue une stratégie employée lorsque les individus dans la population ciblée sont difficiles à atteindre et qu'il n'existe pas de cadre d'échantillonnage connu[11]. Les méthodes d'échantillonnage en fonction des répondants ont été décrites précédemment [11]. Un total de 6 équipes [Yaoundé : 2, Douala : 1, Bertoua : 1, Bamenda : 1, Kribi : 1] a permis le recrutement de 2 255 participantes. Les participantes ont reçu 2 000 francs CFA d'Afrique centrale pour leur participation et 500 francs CFA, approximativement 0,85 USD, pour chaque participante éligible qu'elles ont recrutée, avec un maximum de trois recrues. Les TS ont été recrutées à Yaoundé, Douala, Bertoua, Bamenda et Kribi, entre décembre 2015 et octobre 2016.

Les participantes éligibles étaient des femmes de 18 ans et plus, déclarées femmes à la naissance, et dont le commerce sexuel avait été la première source de revenus au cours des 12 derniers mois. Les femmes ont été incluses dans l'analyse actuelle si elles étaient séropositives au VIH et avaient au moins un enfant en vie au moment de l'étude. Toutes les participantes à l'étude ont donné leur consentement éclairé écrit soit en anglais soit en français, avant de s'engager dans l'étude. L'approbation éthique de l'étude a été obtenue auprès du comité d’éthique de la recherche au Cameroun, ainsi qu'auprès du comité d'examen institutionnel (Institutional Review Board) de la Johns Hopkins School of Public Health.

Après avoir donné leur consentement éclairé écrit, les femmes ont rempli un questionnaire de 45 à 60 minutes soumis par l'enquêteur, pour répondre à des questions liées à

la démographie, à l'histoire de la santé sexuelle et reproductive, aux violations des droits de l'Homme, ainsi qu'à l'utilisation des services de prévention et de traitement pour le VIH. Des prélèvements sanguins et des tests de dépistage du VIH ont été conduits dans le respect des procédures nationales. Pour le dépistage du VIH, toutes les femmes ont reçu un test rapide d'orientation diagnostique : kit de test rapide Alere Determine™ HIV-1/2 Ag/Ab Combo. En l'absence de réaction, le résultat du test pour la participante était enregistré comme négatif au VIH. En cas de réaction, un deuxième test rapide, OraSure OraQuick® HIV- 1/2 était administré. En cas de réaction aux tests OraQuick HIV- 1/2, le résultat du test pour la participante était enregistré comme positif au VIH. En l'absence de réaction, le résultat du test pour la participante était enregistré comme négatif au VIH. Un conseil avant et après le test était fourni comme stipulé dans les directives nationales. Les femmes testées positives au VIH étaient orientées vers un établissement de traitement et de soins pour la suite de la prise en charge.

Résultats

Le premier résultat examiné dans ces analyses a été la proportion des enfants d'une femme soumis à un test de VIH avant l'âge de cinq ans. La proportion des enfants testés a été calculée en prenant le nombre d'enfants testés pour le VIH avant l'âge de cinq ans [“Combien d'enfants avez-vous fait tester pour le VIH avant l'âge de 5 ans ?”] et en le divisant par le nombre total d'enfants vivants qu'une femme indiquait avoir [“Combien avez-vous d'enfants biologiques en vie ?”]. Les deux réponses étaient sur déclaration.

Covariables d'intérêt

La principale variable d'intérêt indépendante était le recours aux SPN durant la dernière grossesse, avec les réponses “oui, a eu recours” ou “non, n'a pas eu recours.” On a demandé aux femmes “La dernière fois que vous étiez enceinte, êtes-vous allée à la clinique pour avoir recours à des soins prénatals pendant votre grossesse ? Il s'agit de soins que vous recevez par des professionnels de santé pendant votre grossesse, afin de s'assurer que vous et votre bébé allez bien et pour promouvoir une grossesse saine.” D'autres covariables d'intérêt incluaient le site de recrutement, l'âge, le niveau d'éducation, les revenus, la durée de l'activité sexuelle

commerciale, les intentions de grossesses futures, la parité, la taille du réseau et la conscience de son statut VIH, tous ces éléments, à part le site de recrutement, ont été documentés sur une base déclarative. Les intentions de grossesses futures ont été triées et on a demandé aux femmes “Prévoyez-vous ou espérez-vous avoir d'autres enfants à l'avenir ?” Pour obtenir la taille des réseaux, on a demandé aux femmes “Combien de travailleuses du sexe avez-vous rencontrées au cours de la semaine passée ?”

Analyses statistiques

Parmi les TS porteuses du VIH et qui avaient au moins un enfant en vie, les caractéristiques ont été comparées par site de recrutement au moyen de la méthode exacte de Fisher. La régression logistique a été utilisée pour examiner les associations entre le recours aux SPN et d'autres indicateurs et la probabilité d'avoir un enfant testé pour le VIH avant l'âge de cinq ans. Les associations brutes et rectifiées ont été examinées. Des indicateurs covariables ont été sélectionnés pour inclusion dans le modèle à multivariable final, sur la base d'une prise en compte à la fois des liens statistiquement significatifs avec le résultat dans les modèles à variable unique ($p<0.10$) et des relations supposées *a priori*. Comme toutes les variables présentaient moins de 1 % de données manquantes, l'analyse de cas complet a été utilisée.

Analyse de sensibilité

Le lien entre le recours aux SPN et la probabilité qu'un enfant ait été testé pour le VIH avant l'âge de cinq ans a également été examiné sur un échantillon restreint de femmes qui avaient des enfants de cinq ans ou moins au moment de l'étude. En se limitant aux femmes avec des enfants de moins de cinq ans, une mesure plus proximale du risque a été évaluée, étant donné que les directives de dépistage évoluent rapidement et que les enfants nés au cours des cinq dernières années sont à même de refléter les tendances actuelles de dépistage.

RÉSULTATS

Sur les 2 255 TS recrutées pour participer à l'étude primaire, 1 889 (84 %) étaient mères, et

environ 24 % [95 % IC : 23 %, 26 %] étaient porteuses du VIH (547/2 255). Sur les 2 255 femmes recrutées pour l'étude primaire, 1 708 ont été exclues de ces analyses car elles n'étaient pas porteuses du VIH. 66 autres porteuses du VIH ont été exclues car elles n'avaient pas d'enfant. Parmi les 481 TS dans ces analyses, 84 % ont recouru à des SPN durant leur dernière grossesse (406/481), et 52 % avaient connaissance de leur séropositivité (251/481). La médiane du nombre d'enfants par femme était de deux (EI : 1-3). Presque 70 % des femmes ont rapporté qu'aucun de leurs enfants n'avait été dépisté pour le VIH avant l'âge de cinq ans (326/481), et 17 (3,5 %) ont rapporté qu'au moins un de leurs enfants avait été diagnostiqué séropositif.

Les caractéristiques des participantes à l'étude selon le site de recrutement sont décrites (**Tableau 1).** La répartition des femmes était relativement égale sur les sites, avec environ un quart des femmes incluses recrutées à Yaoundé. Les femmes recrutées à Douala étaient sensiblement moins susceptibles d'avoir eu recours à des SPN durant leur dernière grossesse, tandis que les femmes recrutées à Bertoua et Bamenda étaient significativement plus susceptibles d'y avoir eu recours ($p<0,001$). L'âge médian était de 35 ans (EI : 30-41). Les femmes plus jeunes étaient plus susceptibles d'avoir été recrutées à Bertoua et Kribi, tandis que les femmes plus âgées avaient été recrutées plus souvent à Bamenda ($p<0,001$). Plus de 45 % des femmes avaient un niveau d'éducation limité à l'école primaire ou moins (217/481). Près de 70 % des femmes ont rapporté gagner 100 000 XAF ou moins par mois, soit environ 6 USD par jour, les femmes à faible revenu étant plus susceptibles d'être recrutées à Bamenda ($p<0,001$). Les femmes ont indiqué se prostituer depuis cinq ans en valeur médiane (EI : 3-10). Environ 20 % des femmes n'ont indiqué aucune méthode de contraception autre que les préservatifs (84/481), celles ayant eu recours aux SPN étant plus susceptibles d'utiliser des contraceptifs longue durée réversibles ($p<0,01$). L'âge médian du dernier-né était de 8 ans (EI : 4-13).

Dépistage dans la petite enfance

Le suivi des soins prénatals était associé à des statistiques accrues de dépistage du VIH chez les enfants avant l'âge de 5 ans (RC : 4,40, 95 % IC 2,01, 9,61) (**Tableau 2**). Avoir été recrutée à Douala (RC : 0,18, 95 % IC 0,09, 0,34) ou Kribi (CR : 0,45, 95 % IC 0,24, 0,84) était associé à une

moindre probabilité d'avoir un enfant testé au VIH avant l'âge de cinq ans, alors qu'avoir été recrutée à Bamenda (CR : 4,20, 95 % IC 2,69, 6,53) était associé à une plus forte probabilité d'avoir un enfant testé au VIH, le tout comparé aux femmes recrutées à Yaoundé. Avoir un niveau d'éducation secondaire ou supérieure, comparé à un niveau d'éducation primaire ou moins (CR : 2,98, 95 % IC 1,42, 6,25), des intentions de grossesse future comparé à aucune intention (RC : 1,52, 95 % IC 1,03, 2,24, et connaître son statut VIH plutôt que l'ignorer (RC : 3,20, 95 % IC 2,91, 4,90) ont tous été des facteurs associés à des statistiques accrues d'avoir un enfant testé pour le VIH avant l'âge de cinq ans dans les modèles à variable unique. Dans le modèle à variables multiples incluant le site de recrutement, le recours aux SPN lors de la dernière grossesse, l'âge, le niveau d'éducation, les intentions de grossesse future et la connaissance de son statut VIH, les variables qui sont apparues statistiquement clairement associées à une probabilité accrue de dépistage dans l'enfance sont le site de recrutement, le recours aux SPN, les intentions de future grossesse, la connaissance de son statut VIH et le niveau d'éducation plus élevé. Comparé à un recrutement à Yaoundé, avoir été recrutée à Bamenda restait significativement associé à des probabilités accrues, alors qu'un recrutement à Douala ou Kribi indiquait une baisse des probabilités.

Analyses de sensibilité

Dans les analyses de sensibilité planifiées limitées au sous-échantillon de femmes avec au moins un enfant ≤5 ans au moment de l'étude (n=180), aucune différence qualitative n'a émergé dans les résultats d'évaluation du lien entre et le recours aux SPN et les chances d'avoir un enfant testé pour le VIH avant l'âge de cinq ans (résultats non illustrés).

DISCUSSION

Ces analyses des résultats sur la santé reproductive et maternelle parmi les mères TS porteuses du VIH et leurs enfants soulignent le besoin de prioriser la santé de la mère par un engagement précoce et continu au niveau du dépistage et des soins. Dans ce groupe de TS porteuses du VIH, plus de la moitié des femmes ne connaissaient pas leur statut infectieux et un peu plus d'une femme sur six a déclaré ne pas avoir eu recours aux SPN lors de sa dernière grossesse. Presque 70 % des TS porteuses du VIH ont rapporté qu'aucun de leurs enfants n'avait

été dépisté pour le VIH avant l'âge de cinq ans, et env. 4 % ont rapporté qu'au moins un de leurs enfants avaient été diagnostiqué séropositif. Le recours aux SPN, en combinaison avec des différences spécifiques au site, un niveau d'éducation plus élevé, des intentions de future grossesse et la connaissance de son statut VIH se sont avérés en forte corrélation avec le dépistage dans la petite enfance.

Malgré le besoin d'un dépistage du VIH parmi les enfants des femmes porteuses du VIH - seulement 30 % des TS incluses dans l'étude ont déclaré au moins un enfant testé pour le VIH avant l'âge de cinq ans - on en sait peu sur l'engagement dans le dépistage et les soins ou sur les résultats de santé des enfants des TS[5]. Les données existantes se concentrent principalement sur les défis de la maternité et sur d'autres vulnérabilités pour la femme, en particulier en lien avec le pouvoir de négociation[12-15]. Dans le sud de la Tanzanie, on a découvert que les travailleuses du sexe qui étaient mères étaient plus susceptibles de prendre plus de clients et d'accepter une plus forte rémunération pour des pratiques sexuelles non protégées[12]. Malgré la connaissance des risques, les femmes se sentaient contraintes pour pouvoir subvenir aux besoins de leurs enfants[12]. Une autre étude dans le sud de l'Inde a également montré que les femmes avec trois ou quatre enfants étaient nettement moins susceptibles d'utiliser le préservatif de manière systématique et plus susceptibles d'avoir des rapports sexuels tarifés non protégés[13]. Malgré ces données, on dispose de peu de chiffres sur le dépistage et les résultats de santé des enfants des TS et sur les stratégies de réduction de la transmission verticale au sein de ce groupe vulnérable.

La connaissance du statut VIH était très faible parmi ces mères, et si l'on considère l'engagement maternel dans le dépistage et les soins dans un contexte plus large, il existe des différences claires entre les TS et d'autres femmes en âge de procréer Par exemple, dans une étude unique considérant une cohorte rétrospective combinée de 1 866 mères et une cohorte prospective de 150 mères au Cameroun, la prévalence totale de l'infection maternelle par le VIH était de 5 %[16]. Même avec une connaissance limitée rapportée des modes de transmission verticale, toutes celles testées positives dans ce groupe de femmes connaissaient leur statut comparé à seulement 52 % des TS de cette étude[16]. Le recours aux SPN parmi

d'autres femmes en âge de procréer au Cameroun s'est avéré assez élevé, avec une étude rapportant que 85 % des femmes avaient eu au moins une consultation de SPN en 2011 – 96 % dans les zones urbaines et 76 % dans les zones rurales - comparé à 84 % des TS de cette étude[17]. Étant donné l'incidence disproportionnée du VIH parmi les TS, 24 % des TS dans cette étude comparé à un peu plus de 5 % parmi les autres femmes en âge de procréer[18], et les bas niveaux constatés concernant la couverture par les SPN et la connaissance du statut VIH de ces femmes, une cible essentielle de l'intervention peut être manquée si l'on ne fait pas des travailleuses du sexe et de leurs enfants une priorité dans les efforts pour accroître la fourniture de services.

Les différences observées selon les sites montrent que considérer les estimations et données nationales globalement peut ne pas donner une image précise des disparités concernant la santé maternelle et l'engagement dans les soins en fonction de la région. Des différences dans les estimations régionalisées des indicateurs de santé infantile ont été observées précédemment, ce qui conforte les conclusions que les taux de dépistage dans la petite enfance peuvent dépendre des régions[19]. Dans cette étude parmi les TS, en comparaison avec Yaoundé, des probabilités inférieures de dépistage dans la petite enfance ont été observées à Douala et Kribi. Dans ces mêmes zones, une enquête démographique et sanitaire de plus grande ampleur a détecté une prévalence supérieure d'enfants de moins de cinq ans classés comme mal nourris et anémiques[19]. Les TS à Bamenda ont rapporté de hauts niveaux de recours aux SPN et au dépistage dans la petite enfance. Ce succès dans la région Nord-Ouest du pays peut refléter la récente amplification des programmes de PTME [18]. Un accroissement du financement a permis d'augmenter la couverture géographique et l'étendue des programmes de PTME, avec des efforts de mobilisation des communautés, de créer un groupe de travail technique sur la PTME, d'avoir plus de personnel et d'engager d'autres efforts [18]. Selon un rapport, les services de PTME soutenus par le PEPFAR ont permis d'augmenter le recours aux SPN et la proportion de femmes enceintes soumises à un test du VIH[1]. L'élaboration et la fourniture d'estimations régionalisées et d'autres recherches visant à

comprendre les différences régionales seront cruciales pour fournir des interventions ciblées et efficaces, à la fois pour les TS et pour la population générale.

Ces analyses présentent des limitations. L'échantillonnage en fonction des répondants n'a pas été corrigé au moment d'analyser le sous-groupe des mères séropositives dans la population de l'étude, ainsi les chaînes de recrutement ont été interrompues et les connexions requises pour comprendre les liens de réseau et appliquer des ajustements n'ont pas été effectuées. On a demandé aux femmes si elles avaient eu recours aux SPN durant leur dernière grossesse mais on ne peut pas dire si une femme a eu recours aux SPN pour chacune de ses autres grossesses et quel est l'impact sur la santé d'un enfant en particulier. Malgré cette limitation, le recours aux SPN pendant la dernière grossesse indique l'engagement d'une femme dans les soins pendant la grossesse, une période critique pour éviter la transmission verticale. De plus, les premières analyses ne considèrent pas les effets de la modification des directives de dépistage. Toutefois, nous avons tenté de comprendre ces effets en limitant l'échantillon des analyses de sensibilité aux femmes ayant eu des enfants au cours des cinq dernières années, et aucune différence notable n'est apparue au moment de considérer les relations d'intérêt primaire. En outre, vu la structure des questions, nous ne pouvons affirmer si les enfants décédés avaient été testés et dans quelle mesure la mortalité est liée au VIH.

CONCLUSIONS

La prévalence élevée du VIH chez la mère et le faible niveau de connaissance du statut VIH dans ce groupe de TS soulignent le besoin d'envisager différentes stratégies pour renforcer les interventions actuelles de PTME. En considérant que de nombreuses TS sont mères et que la promotion de la santé de la mère par un engagement précoce dans le dépistage et les soins pour elle et les enfants peuvent améliorer les résultats sur la santé maternelle et infantile. Il sera important d'intégrer des services spécifiques à l'enfant, y compris le dépistage du VIH durant la petite enfance, dans les services aux TS existants, et les différences spécifiques aux sites seront à considérer pour le choix d'interventions appropriées dans le contexte. En raison des obstacles aux soins, l'accueil des femmes difficiles d'accès dans les programmes nationaux

de PTME, y compris la sensibilisation et la réduction de la stigmatisation des soignants envers la prostitution, la promotion du recours aux SPN et au dépistage du VIH parmi les TS, ainsi qu'une orientation active vers les SPN par les organisations communautaires travaillant avec les TS, peuvent aider à réduire les lacunes dans l'accès et le recours à la PTME. Multiplier les intervenants en approche communautaire et les chargés de dossiers dans les organisations communautaires peut contribuer à une meilleure articulation avec les soins dans les centres de santé communautaires. Les services de SPN pour les femmes à risque peuvent être renforcés grâce à des services de dépistage du VIH intensifiés et des informations sur la prévention de la transmission verticale, afin d'améliorer les résultats de santé pour la mère et l'enfant.

Liste des abréviations

VIH : virus de l'immunodéficience humaine

TS : travailleuses du sexe

SPN : soins prénatals

PTME : prévention de la transmission de la mère à l'enfant

OMS : Organisation mondiale de la Santé

EBCI : enquête biocomportementale intégrée

XAF : franc CFA d'Afrique centrale

USD : dollar américain

Concernant ce supplément

Cet article a été publié comme partie de l'ouvrage de *BMC Public Health*, Volume 19 Supplement 1, 2019: Effective Integration of Sexual Reproductive Health and HIV Prevention, Treatment, and Care Services across sub-Saharan Africa: Where is the evidence for program implementation?
Le supplément a été publié conjointement entre *Reproductive Health* et *BMC Public Health.* L'intégralité du contenu, avec les versions en français portugais et anglais, est disponible en ligne :
https://bmcpublichealth.biomedcentral.com/articles/supplements/volume-19-supplement-1
et
https://reproductive-health-journal.biomedcentral.com/articles/supplements/volume-16-supplement-1

Déclarations

Approbation éthique et accord de participation

Toutes les participantes à l'étude ont donné leur consentement éclairé écrit soit en anglais soit en français, avant de s'engager dans l'étude. L'approbation éthique de l'étude a été obtenue auprès du comité d'éthique de la recherche au Cameroun, ainsi qu'auprès du comité d'examen institutionnel (Institutional Review Board) de la Johns Hopkins School of Public Health.

Accord de publication

Non applicable.

Disponibilité des données et matériels

Les données utilisées et/ou analysées durant l'étude actuelle sont disponibles auprès de l'auteur-ressource sur demande raisonnable.

Conflits d'intérêts

Les auteurs déclarent ne pas avoir de conflits d'intérêts.

Financement

L'étude a été rendue possible grâce au financement par USAID | CHAMP Project, Task Order No.2, est financée par l'U.S. Agency for International Development (Agence des États-Unis pour le développement international) aux termes du contrat n° AID-624-A-14-00003, à compter d'avril 2014, et soutenue par le President's Emergency Plan for AIDS Relief (Plan d'urgence présidentiel américain pour la lutte contre le SIDA). La continuité de la prévention, des soins et des traitements (CoPST) des populations les plus à risque de VIH/SIDA (CHAMP) est menée par CARE International et gérée par CARE Cameroun. Cette recherche a été facilitée par l'infrastructure et les ressources fournies par le Johns Hopkins University Center for AIDS Research, un programme financé par les National Institutes of Health (1P30AI094189), qui est soutenu par les instituts et centres participants et co-

financeurs des NIH suivants : NIAID, NCI, NICHD, NHLBI, NIDA, NIMH,NIA, FIC, NIGMS, NIDDK et OAR. Le contenu est de la seule responsabilité des auteurs et n'illustre pas nécessairement les opinions officielles du NIH.

Le supplément de la revue est rendu possible grâce au soutien généreux du peuple américain via la United States Agency for International Development (USAID) en partenariat avec le Fonds des Nations unies pour la population (FNUAP) et le Programme commun des Nations Unies sur le VIH/SIDA (ONUSIDA).

Les opinions exprimées dans la présente publication sont celles des auteurs et ne reflètent pas nécessairement les politiques officielles de l'USAID, du FNUAP ou de l'ONUSIDA, la mention des dénominations de ministères ou d'organismes n'implique pas non plus l'aval du gouvernement américain, du FNUAP ou de l'ONUSIDA.

Contributions des auteurs

AR était responsable de l'analyse et de l'interprétation des modèles statistiques principaux et a pris le rôle principal dans la rédaction du manuscrit. **SS** a fourni des commentaires précieux sur le contenu et la structure du manuscrit, et une orientation dans la mise au point de modèles statistiques et dans l'élaboration de la question de recherche principale. **SB** a fourni des commentaires précieux sur le contenu et la structure du manuscrit et dans l'interprétation des résultats. **AB** a fourni des commentaires précieux sur le contenu et la structure du manuscrit, et une orientation dans la mise au point de modèles statistiques et dans l'élaboration de la question de recherche principale. **GF** a fourni des commentaires précieux sur le contenu et la structure du manuscrit et dans l'interprétation des résultats. **FN** a fourni des commentaires précieux sur le contenu et la structure du manuscrit et dans l'interprétation des résultats. **IN** a fourni des commentaires précieux sur le contenu et la structure du manuscrit et dans l'interprétation des résultats. **DL** a fourni des commentaires précieux sur le contenu et la structure du manuscrit et dans l'interprétation des résultats.

ACB a fourni des commentaires précieux sur le contenu et la structure du manuscrit et dans l'interprétation des résultats. **ON** a fourni des commentaires précieux sur le contenu et la structure du manuscrit et dans l'interprétation des résultats. **SB** a fourni des commentaires précieux sur le contenu et la structure du manuscrit, et une orientation dans la mise au point de modèles statistiques et dans l'élaboration de la question de recherche principale. **Tous les auteurs ont lu et approuvé le manuscrit final.**

RÉFÉRENCES

1. PEPFAR, *Cameroon: Country Operational Plan*. 2014.
2. UNAIDS. *Cameroon Demographic Health Survey (DHS) 2011: HIV Fact Sheet*. Available from: http://preview.dhsprogram.com/pubs/pdf/HF42/HF42.pdf. Date accessed January 7th 2018.
3. *Rapport Annuel 2014 des activites de lutte contre le VIH/SIDA et les IST au Cameroun*. National AIDS Control Committee (NACC) --Cameroon.
4. Newell, M., et al., *Mortality of infected and uninfected infants born to HIV-infected mothers in Africa: a pooled analysis.* Lancet, 2004. **364**(9441): p. 1236-43.
5. UNAIDS. *Global Plan Country Fact Sheet: Cameroon*. 2015; Available from: http://www.unaids.org/sites/default/files/media/documents/UNAIDS_GlobalplanCountryfactsheet_cameroon_en.pdf. Date accessed February 1st 2018.
6. Schwartz, S. and S. Baral, *Fertility-related research needs among women at the margins.* Reprod Health Matters, 2015. **23**(45): p. 30-46.
7. *Mapping and size estimation of female sex workers in Cameroon to inform HIV program design and implementation*. 2016, World Bank.
8. Willis, B., S. Onda, and H.M. Stoklosa, *Causes of maternal and child mortality among Cambodian sex workers and their children: a cross sectional study.* BMC Public Health, 2016. **16**(1): p. 1176.
9. UNAIDS. *On the fast-track to an AIDS-free generation*. 2016; Available from: http://www.unaids.org/sites/default/files/media_asset/GlobalPlan2016_en.pdf. Date accessed January 12th 2018.
10. Bonono, R.C. and P. Ongolo-Zogo, *Optimizing the use of antenatal care services in Cameroon*, in *Centre for Development of Best Practices in Health -- Central Hospital*. 2012: Yaounde, Cameroon.
11. Heckathorn, D., *Respondent-driven sampling: a new approach to the study of hidden populations.* Social Problems, 1997. **44**(2).
12. Beckham, S.W., et al., *'If you have children, you have responsibilities': motherhood, sex work and HIV in southern Tanzania.* Cult Health Sex, 2015. **17**(2): p. 165-79.

13. Reed, E., et al., *Motherhood and HIV risk among female sex workers in Andhra Pradesh, India: the need to consider women's life contexts.* AIDS Behav, 2013. **17**(2): p. 543-50.
14. Servin, A.E., et al., *Vulnerabilities faced by the children of sex workers in two Mexico-US border cities: a retrospective study on sexual violence, substance use and HIV risk.* AIDS Care, 2015. **27**(1): p. 1-5.
15. Castaneda, X., et al., *Sex masks: the double life of female commercial sex workers in Mexico City.* Cult Med Psychiatry, 1996. **20**(2): p. 229-47.
16. Sama, C., et al., *Prevalence of maternal HIV infection and knowledge of mother-to-child transmission of HIV and its prevention among antenatal care attendees in a rural area in northwest Cameroon.* PLOS One, 2017.
17. *Enquête Démographique et de Santé et a Indicateurs Multiples IDS-MICS*. 2011, Institut National de La Statistique.
18. Bonje, E.K., E.M. Khan, and L. Miller, *12 years of implementing PMTCT services in Cameroon*. 2012, Elizabeth Glaser Pediatric AIDS Foundation.
19. *2011 Cameroon Demographic and Health Survey and Multiple Indicators Cluster Survey (DHS-MICS): HIV prevalence*. 2012, Cameroon National Institute of Statistics: Calverton.

Tableau 1. Caractéristiques des travailleuses du sexe porteuses du VIH par site de recrutement au Cameroun, 2015-2016 (n=481)

	**Total** n= 481	**Yaoundé** n=120	**Douala** n=122	**Bertoua** n=61	**Bamenda** n=101	**Kribi** n=77	**valeur p**
	n (%)	n (%)	n (%)	n (%)	n (%)	n (%)	
**Recours aux SPN à la dernière grossesse**							**<0,001**
Non, pas de recours	75 (15,6 %)	20 (16,7 %)	35 (28,7 %)	4 (6,6 %)	3 (3,0 %)	13 (16,9 %)	
Recours	406 (84,4 %)	100 (83,3 %)	87 (71,3 %)	57 (93,4 %)	98 (97,0 %)	64 (83,1 %)	
**Âge, années**							
18-24	43 (8,9 %)	6 (5,0 %)	6 (4,9 %)	15 (24,6 %)	6 (5,9 %)	10 (13,0 %)	**<0,001**
25-34	182 (37,9 %)	46 (38,3 %)	39 (32,0 %)	28 (45,9 %)	29 (28,7 %)	40 (52,0 %)	
35+	256 (53,2 %)	68 (56,7 %)	77 (63,1 %)	18 (29,5 %)	66 (65,4 %)	27 (35,0 %)	
**Niveau d'éducation[a]**							
Primaire ou moins	217 (45,2 %)	38 (31,7 %)	60 (49,2 %)	22 (36,7 %)	68 (67,3 %)	29 (37,7 %)	**<0,001**
Secondaire partiel	237 (49,4 %)	74 (61,7 %)	57 (46,7 %)	36 (60,0 %)	25 (24,8 %)	25 (58,4 %)	
Secondaire ou plus	26 (5,4 %)	8 (6,6 %)	5 (4,1 %)	2 (3,3 %)	8 (7,9 %)	3 (3,9 %)	
**Revenus mensuels**							
≤100 000 XAF	329 (68,4 %)	87 (72,5 %)	47 (38,5 %)	39 (63,9 %)	99 (98,0 %)	57 (74,0 %)	**<0,001**
>100 000 XAF	152 (31,6 %)	33 (27,5 %)	75 (61,5 %)	22 (36,1 %)	2 (2,0 %)	20 (26,0 %)	
**Années de prostitution**	109 (22,7 %)	24 (20,0 %)	24 (19,6 %)	21 (34,4 %)	10 (9,9 %)	30 (39,0 %)	**<0,001**
0-2 ans	228 (47,4 %)	60 (50,0 %)	70 (57,4 %)	20 (47,5 %)	35 (34,7 %)	34 (44,1 %)	
3-9 ans	144 (29,9 %)	36 (30,0 %)	28 (23,0 %)	11 (18,0 %)	56 (55,4 %)	13 (16,9 %)	
10 ans et +							
**Connaissance du statut VIH**							
Statut connu	230 (47,8 %)	56 (46,7 %)	49 (40,2 %)	36 (59,0 %)	34 (33,7 %)	55 (71,4 %)	**<0,001**
Statut inconnu	251 (52,2 %)	64 (53,3 %)	73 (59,8 %)	25 (41,0 %)	67 (66,3 %)	22 (28,6 %)	

**Nombre d'enfants en vie**							
Un	132 (27,4 %)	28 (23,3 %)	43 (35,3 %)	13 (21,3 %)	21 (20,8 %)	27 (35,0 %)	**0,02**
Deux	145 (30,2 %)	47 (39,2 %)	28 (23,0 %)	19 (31,2 %)	27 (26,7 %)	24 (31,2 %)	
Plus de deux	204 (42,4 %)	45 (37,5 %)	51 (41,7 %)	29 (47,5 %)	53 (52,5 %)	26 (33,8 %)	
**Intentions de grossesses futures**							
Aucune	267 (55,5 %)	55 (45,8 %)	81 (66,4 %)	36 (59,0 %)	68 (67,3 %)	27 (35,1 %)	**<0,001**
Veut d'autres enfants	214 (44,5 %)	65 (54,2 %)	41 (33,6 %)	25 (41,0 %)	33 (32,7 %)	50 (64,9 %)	
**Taille du réseau**[b]							
Moyenne	13,4	14,4	20,3	13,8	9,7	5,3	**<0,001**
Écart type	22,3	25,5	29,7	19,8	12,5	6,3	

[a]Données manquantes sur l'éducation (n=1). Les résultats en gras représentent les résultats statistiquement significatifs au moins au niveau p<0,05.

[b]Différences significatives calculées avec le test de Bartlett des variances identiques. Pour obtenir la taille des réseaux, on a demandé aux femmes “Combien de travailleuses du sexe avez-vous rencontrées au cours de la semaine passée ?”

Tableau 2. Rapports de cotes bruts et corrigés des enfants porteurs du VIH et dépistés avant l'âge de cinq ans parmi les travailleuses du sexe porteuses du VIH au Cameroun, 2015-2016 (n=477)[a]

	Rapports de cotes [95 % IC]	Rapports de cotes corrigés [95 % IC]
**Site de recrutement**		
Yaoundé	RÉF.	RÉF.
Douala	**0,18 [0,09, 0,34]*** **	**0,20 [0,10, 0,41]*** **
Bertoua	1,17 [0,66, 2,05]	1,02 [0,50, 2,06]
Bamenda	**4,20 [2,69, 6,53]*** **	**2,34 [1,30, 4,22]** **
Kribi	**0,45 [0,24, 0,84]* **	**0,46 [0,22, 0,98]* **
**Recours aux soins prénatals**		
Pas de recours	RÉF.	RÉF.
Recours durant la dernière grossesse	**4,40 [2,01, 9,61]*** **	**2,12 [1,02, 4,55]* **
**Âge**		
18-24	RÉF.	RÉF.
25-34	1,13 [0,76, 1,67]	0,71 [0,31, 1,61]
35+	0,79 [0,54, 1,16]	0,56 [0,24, 1,30]
**Éducation**		
Primaire ou moins	RÉF.	RÉF.
Secondaire partiel	0,75 [0,51, 1,10]	0,92 [0,57, 1,49]
Secondaire ou plus	**2,98 [1,42, 6,25]**	**2,17 [1,01, 4,71]* **
**Années de prostitution**		
0-2 ans	RÉF.	
3-9 ans	0,90 [0,61, 1,33]	
10 ans et +	1,34 [0,89, 2,03]	
**Intentions de grossesses**		
Aucune intention	RÉF.	RÉF.
A des intentions	**1,52 [1,03, 2,24]* **	**1,89 [1,16, 3,08]* **
**Nombre d'enfants en vie**		
Un	RÉF.	

Deux<br>Plus de deux	0,84 [0,55, 1,29]<br>1,09 [0,74, 1,60]	
**Connaissance du statut VIH**<br>Statut inconnu<br>Statut VIH connu	 RÉF.<br>**3,20 [2,91, 4,90]*** **	 RÉF.<br>**3,70 [2,30, 5,93]*** **
**Taille du réseau**	0,99 [0,97, 1,01]	

Les rapports de cotes ont été estimés par régression logistique. [a]Cinq femmes ont été exclues de l'analyse en raison de données manquantes sur la proportion d'enfants dépistés avant l'âge de cinq ans (n=4) ou leur plus haut niveau d'éducation (n=1). *Statistiquement significatif à p<0,05 ; **p<0,01 ; ***p<0,001. Les résultats en gras représentent les résultats statistiquement significatifs au moins au niveau p<0,05.
