# Supplementary material for: Predictors of early childhood HIV testing among children of sex workers living with HIV in Cameroon
Source: BMC Public Health. 2019 May 29;19(Suppl 1):602. doi: 10.1186/s12889-019-6812-3 (PMC6538542; doi:10.1186/s12889-019-6812-3)
Supplement: Supplementary file 2 — Translation of the abstract of this article into Portuguese (PDF 101 kb) [file 12889_2019_6812_MOESM2_ESM.pdf]

BMC Public Health 2019, 19 (Suppl 1) [translated from the original publication]

Indicadores da necessidade de realizar testes do VIH durante a primeira infância dos filhos das trabalhadoras do sexo que vivem com VIH nos Camarões

Amrita Rao[1], Sheree Schwartz[1], Serge C. Billong[2], Anna Bowring[1], Ghislaine Fouda[3], Flavien Ndonko[3], Iliassou Njindam[1], Daniel, Levitt[4], Anne-C. Bissek[5,6], Oudou Njoya[5], Stefan Baral[1]

[1]Johns Hopkins Bloomberg School of Public Health, Epidemiology, Baltimore, Maryland, United States
[2]University of Yaoundé, Yaoundé, Cameroon
[3]CARE Cameroun, Yaoundé, Cameroon
[4]CARE USA, New York, New York, United States
[5]University of Yaoundé, Faculty of Medicine and Biomedical Sciences, Yaoundé, Cameroon
[6]Ministry of Public Health, Division of Operational Research in Health, Yaoundé, Cameroon

Autor correspondente:
Amrita Rao, ScM, Department of Epidemiology, Johns Hopkins Bloomberg School of Public Health, 615 N. Wolfe St. Baltimore, MD, 21205
E-mail: arao24@jhu.edu

Endereço de e-mail do coautor:
Sheree Schwartz sschwartz@jhu.edu, Serge Billong sergebillong@yahoo.fr, Anna Bowring abowrin1@jhu.edu, Ghislane Fouda fouda@carecameroun.org, Flavien Ndonko ndonko@carecameroun.org, Iliassou Mfochive Njindam imfochi1@jhu.edu, Daniel Levitt daniel_levitt@me.com, Anne Bissek annezkbissek@yahoo.fr, Oudou Njoya oudou_nj@yahoo.fr, Stefan Baral sbaral@jhu.edu

RESUMO

Introdução: Apesar dos progressos recentes, existem lacunas no acesso e na adoção do programa de prevenção da transmissão vertical do VIH nos Camarões. As trabalhadoras do sexo, muitas das quais são mães, são afetadas de forma desproporcionada pelo VIH e enfrentam obstáculos específicos aos testes do VIH, bem como acesso ao tratamento. A realização de testes em bebés expostos ao VIH é essencial na monitorização de ocorrências de infeção e na intervenção oportuna. Este estudo investiga o nível dos testes realizados durante a primeira infância e as associações entre o recurso aos serviços de cuidados pré-natais e outros fatores e a realização de testes do VIH durante a primeira infância dos filhos das trabalhadoras do sexo nos Camarões.

Métodos: As trabalhadoras do sexo foram recrutadas para participar num inquérito biocomportamental integrado nos Camarões entre dezembro de 2015 e outubro de 2016. As mulheres incluídas nestas análises tinham de viver com o VIH e ter pelo menos um filho vivo. Tanto o modelo de regressão logística univariada como o modelo de regressão logística multivariada foram utilizados para analisar indicadores da necessidade de uma criança ser testada para despiste do VIH antes dos cinco anos de idade.

Resultados: Um total de 481/2255 das trabalhadoras do sexo foram elegíveis para estas análises, uma vez que eram seropositivas (VIH) e tinham pelo menos um filho vivo aquando da realização do estudo. As mulheres incluídas nestas análises tinham uma idade média de 35 anos (IQR 30-41). Cerca de 70% indicaram que os filhos nunca tinham sido submetidos à despistagem do VIH antes dos cinco anos de idade (326/481) e 3,5% (17/481) afirmaram que um ou mais filhos tinham sido diagnosticados com VIH. Os fatores de recurso aos serviços de cuidados pré-natais (OR ajustada de 2,12, IC 95%: [1,02; 4,55]), o conhecimento do seu estatuto de portadora do VIH (OR de 3,70 [2,30; 5,93]), o desejo de gravidez (OR ajustada de 1,89 [1,16; 3,08]) e o nível de educação superior (OR ajustada de 2,17 [1,01; 4,71]) foram todos associados de forma independente a um aumento da probabilidade dos filhos destas mulheres realizarem testes de despistagem do VIH antes dos cinco anos de idade. Também foram observadas diferenças regionais nos testes realizados durante a primeira infância.

Conclusão: A transmissão vertical do VIH continua a constituir um desafio nos Camarões e a realização de testes aos filhos das trabalhadoras do sexo que vivem com o VIH foi muito baixa. O recurso aos serviços de cuidados de saúde pré-natais e a promoção da saúda das mães estão associados a uma maior despistagem do VIH nas crianças. Para mulheres com um risco elevado de contrair VIH e um nível inferior de utilização do sistema de saúde, as estratégias para promover e assegurar o recurso a serviços de cuidados pré-natais são essenciais para a saúde das mães e crianças.

Palavras-chave: Trabalhadoras do sexo feminino, Transmissão vertical do VIH, Testes do VIH durante a primeira infância, Recurso a serviços de cuidados pré-natais, Camarões

Sobre este suplemento

Este resumo foi publicado como parte da revista científica *BMC Public Health*, Volume 19, Suplemento 1, 2019: Integração Eficaz dos Serviços de Saúde Sexual e Reprodutiva e de Prevenção, Cuidados e Tratamento do VIH na África Subsariana: Onde estão as provas da implementação do programa?
O suplemento foi publicado como uma colaboração entre as revistas científicas *Reproductive Health* e *BMC Public Health.* O conteúdo integral do suplemento, incluindo as versões em francês, português e inglês, estão disponíveis online:
https://bmcpublichealth.biomedcentral.com/articles/supplements/volume-19-supplement-1
e
https://reproductive-health-journal.biomedcentral.com/articles/supplements/volume-16-supplement-1
